# Overexpression of myeloid differentiation protein 88 in mice induces mild cardiac dysfunction, but no deficit in heart morphology

**DOI:** 10.1590/1414-431X20154794

**Published:** 2015-11-27

**Authors:** W. Chen, Z. Huang, X. Jiang, C. Li, X. Gao

**Affiliations:** 1 Institute for Cardiovascular Science & Department of Cardiovascular Surgery of the First Affiliated Hospital, Soochow University, Suzhou, Jiangsu, China; 2 MOE Key Laboratory of Model Animal for Disease Study, Model Animal Research Center, Nanjing University, Nanjing, Jiangsu, China; 3 Jiangsu Province Key Laboratory of Gastrointestinal Nutrition and Animal Health, College of Animal Science and Technology, Nanjing Agriculture University, Nanjing, Jiangsu, China; 4 Department of Surgery, East Tennessee State University, Johnson City, TN, USA

**Keywords:** Cardiac dysfunction, Cardiac remodeling, Transgenic mice, Myeloid differentiation protein 88

## Abstract

Cardiac remodeling involves changes in heart shape, size, structure, and function after injury to the myocardium. The proinflammatory adaptor protein myeloid differentiation protein 88 (MyD88) contributes to cardiac remodeling. To investigate whether excessive MyD88 levels initiate spontaneous cardiac remodeling at the whole-organism level, we generated a transgenic MyD88 mouse model with a cardiac-specific promoter. MyD88 mice (male, 20-30 g, n=∼80) were born at the expected Mendelian ratio and demonstrated similar morphology of the heart and cardiomyocytes with that of wild-type controls. Although heart weight was unaffected, cardiac contractility of MyD88 hearts was mildly reduced, as shown by echocardiographic examination, compared with wild-type controls. Moreover, the cardiac dysfunction phenotype was associated with elevation of *ANF* and *BNP* expression. Collectively, our data provide novel evidence of the critical role of balanced MyD88 signaling in maintaining physiological function in the adult heart.

## Introduction

Cardiac remodeling refers to changes in size, shape, structure, and function of the heart after injury to the myocardium (1). This injury may be due to myocarditis (2), acute myocardial infarction (3), chronic hypertension (4), and congenital heart diseases (5,6). During cardiac remodeling, a series of histopathological and structural changes occur, finally resulting in reduced stroke volume and decreased contractile function. There are multiple mechanisms underlying cardiac remodeling, one of which is mediated by inflammatory signaling molecules (7 8 9).

Toll-like receptors (TLRs) are pattern recognition receptors that recognize exogenous pathogen-associated molecular patterns to activate the host innate immune defense (10). In addition to the primary role of TLRs in response to microbial infections, TLRs can also recognize endogenous ligands and mediate cardiac remodeling (7-9). TLRs use five signaling adaptors to mediate receptor activation to downstream signal transduction (11). Importantly, all TLRs, except for TLR3, signal through myeloid differentiation factor 88 (MyD88) for activation of nuclear factor-κB, leading to the production of inflammatory mediators (12).

Although MyD88 was originally identified as a myeloid-differentiation marker (13), MyD88 is typically known to play an essential role in the innate immune response (14). Generation of MyD88-deficient mice has revealed new and important insights into the function of MyD88 in cardiac remodeling of several cardiomyopathy diseases. MyD88-knockout mice are protected from experimental autoimmune myocarditis (15), Coxsackievirus B3-induced myocarditis (16), and endotoxin-induced cardiomyopathy (17 18 19). Specifically, Feng et al. (19) demonstrated that cardiac MyD88 mainly contributes to myocardial inducible nitric oxide synthase induction. Interestingly, our previous studies using cardiac-specific dominant negative MyD88 (dnMyD88) transgenic mice demonstrated that uncontrolled MyD88 signaling triggers dilated cardiomyopathy and spontaneous heart failure (20). The above-mentioned results indicate the comprehensive role of MyD88 in maintenance of heart function. However, whether enforced expression of MyD88 initiates spontaneous cardiac remodeling at the whole-organism level remains unclear.

To determine whether excessive MyD88 induces *in vivo* cardiac remodeling at baseline, we targeted MyD88 expression in the myocardium of transgenic mice via an α-myosin heavy chain (α-MHC) promoter. Each transgenic line was viable and demonstrated a significant elevation in basal MyD88 expression. Our data suggested that overexpression of MyD88 did not trigger obvious abnormality in heart morphology, but promoted mild cardiac dysfunction at baseline, with an elevation in heart failure marker expression.

## Material and Methods

### Generation of transgenic mice

Mice (male, 20-30g, n=∼80) were maintained in an Association for Assessment and Accreditation of Laboratory Animal Care International-credited specific pathogen-free animal facility of the Model Animal Research Center (MARC) of Nanjing University. All animal protocols were approved based on the local ethics legislation for animal experimentation. Animal welfare and experimental procedures were conducted with the approval of the Institutional Animal Care and Use Committee of MARC.

The transgenic construct contains the α-MHC promoter (21), Flag-tagged MyD88 cDNA, and a human growth hormone poly-adenylation signal. MyD88 cDNA was amplified and ligated into the *Sal*I and *Hind*III sites of the murine α-MHC promoter expression vector. The encoded MyD88 protein has the Flag tag at the N-terminal. Transgenic mice were generated through pronuclear microinjection of one-cell embryos from the C57BL/6J×CBA F1 hybrid (The Jackson Laboratory, USA). Positive founders were identified by PCR and backcrossed to wild-type (WT) C57BL/6J mice. Genotyping was performed by PCR analyses with the primer sets 5-TTTATCTGCTACTGCCCCAACG-3 (located in exon 3) and 5-CTGGGAAAGTCCTTCTTCATCG-3 (located in exon 5), which were designed to amplify a 711-bp fragment from endogenous MyD88 and a 308-bp fragment from the transgenic MyD88 construct.

### Cytoplasmic protein extraction and western blotting

Myocardial tissue proteins were extracted using 10 mmol/L HEPES, pH 7.9, 10 mmol/L KCl, 0.1 mmol/L EDTA, 0.1 mmol/L EGTA, 1 mmol/L DTT, and protease inhibitors. Protein concentrations were determined by the bicinchoninic acid assay (Pierce Chemical Co., USA). Equal amounts of protein samples were subjected to western blotting as described previously (22,23). Immunoreactivity was revealed with an enhanced chemiluminescent substrate for peroxidase (SuperSignal West Pico substrate, Pierce Chemical Co., USA). Flag antibody was purchased from Sigma (USA).

### Histological examination

A single 3-mm slice of each heart was taken at a similar anatomical location. Sliced tissues were then immersion-fixed in 4% buffered paraformaldehyde and embedded in paraffin (Sigma-Aldrich, USA) for preparation of tissue sections. Serial 5-μm heart sections from each group were analyzed. Hematoxylin and eosin (H&E) staining was subsequently performed on dewaxed sections.

### Semi-quantitative RT PCR

Total RNA was extracted and reverse-transcribed to cDNA using the PrimeScript2 RT-PCR Kit (Takara, Japan). Semi-quantitative RT PCR was performed to examine mRNA expression of atrial natriuretic factor (*ANF*) (5-CGGTGTCCAACACAGATCTGAT-3 and 5-GGCTCCAATCCTGTCAATCCTAC-3) and brain natriuretic peptide (*BNP*) (5-CGAGACAAGGGAGAACACG-3 and 5-CCAAAGCAGCTTGAGATATGT-3). Amplification of *tubulin* (5-TCCATCCACGTCGGCCAGGCT-3 and 5-GTAGGGCTCAACCACAGCAGT-3) served as an input control of cDNA templates.

### Echocardiography

Mice were lightly anesthetized and then their heart function was analyzed using a GE Vingmed Vivid 7 ultrasound scanner (GE Healthcare, USA) with the GE i13L epicardial probe (linear phased array). Echocardiographic measurements were performed in M-mode by an echocardiographer who was blinded to the experimental design as described previously (20).

### Statistical analysis

Statistical analysis was performed with the Graphpad Prism 5 software (Graphpad, USA). The P values were based on the two-tailed Student’s *t*-test and data are reported as means±SE. The null hypothesis was rejected for P values less than 0.05 with the two-tailed test.

## Results

### Generation and characterization of MyD88 transgenic mice

**Figure 1:**
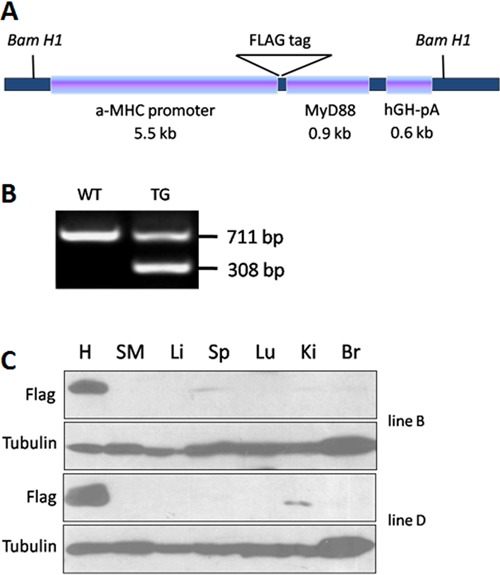
Generation of cardiac-specific transgenic (TG) mice overexpressing MyD88. *A*, Construction map of MyD88 TG mice. MHC: myosin heavy chain; MyD88: myeloid differentiation factor 88; hGH-pA: human growth hormone poly A signal; WT: wild-type. *B*, PCR of genomic DNA from mouse tails was performed for genotyping. A 711-bp fragment from the genomic MyD88 allele and a 308-bp fragment from the transgenic Flag-MyD88 allele was PCR-amplified with the genotyping primers. *C*, Tissue-specific expression of the transgene in MyD88 TG mice was performed with anti-Flag antibody. H: heart; SM: skeletal muscle; Li: liver; Sp: spleen; Lu: lungs; Ki: kidney; Br: brain.

In this study, we targeted Flag-tagged MyD88 expression to the myocardium in transgenic mice using the α-MHC promoter (Figure 1A). The α-MHC promoter directs high-level gene expression specifically in the myocardium after birth (24). The encoded MyD88 protein had the Flag tag at the N-terminal. Four independent transgenic founder lines were established. MyD88 mice were born at the expected Mendelian ratio. Among these independent lines, lines B and D were selected for further analysis based on comparable transgene expression levels, viability, and germline transmission. WT littermates were used as controls in all experiments. PCR of genomic DNA from mouse tails was performed to verify the expected genotypes (Figure 1B). A high level of transgene expression was observed in the MyD88 transgenic (TG) heart of lines B and D (Figure 1C). Weak MyD88 transgene expression was also detected in the spleen of line B and in the kidneys of line D (Figure 1C).

### MyD88 TG mice did not display any abnormality in heart morphology

**Figure 2:**
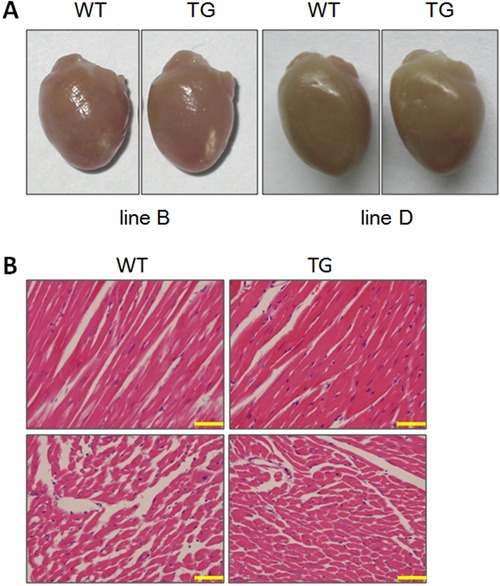
Histological analysis of MyD88 transgenic (TG) hearts. *A*, Gross appearance of the isolated wild-type (WT) and TG heart. *B*, Microscopic view of H&E-stained left ventricular sections from MyD88 TG and WT mice. Scale bar: 2 μm.

Disease progression of human heart failure causes a marked increase in cardiac chamber volume (25). WT and MyD88 TG mice were euthanized at 5 months of age for analysis of cardiac anatomy and histology. As shown in Figure 2A, MyD88 TG hearts showed similar morphology to WT hearts. We performed further H&E staining on serial tissue sections (Figure 2B). No abnormalities in cell size and morphology were observed in left ventricles of MyD88 TG hearts.

**Figure 3:**
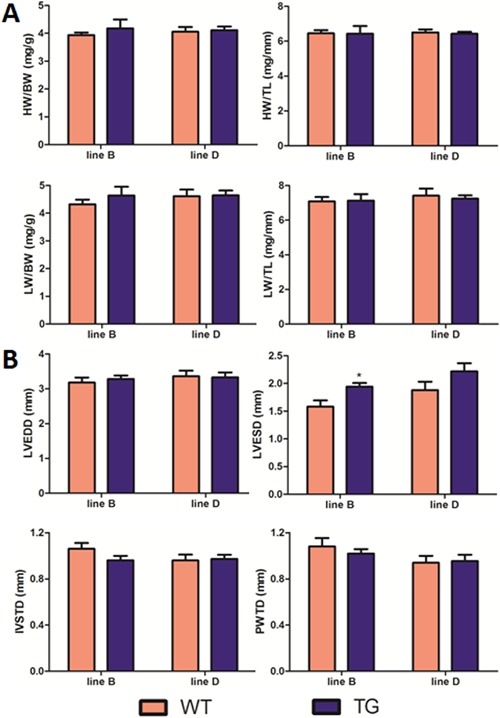
MyD88 transgenic (TG) mice did not show an increased heart size. Data are reported as means ± SE. *A*, Relative heart weights (HW/BW and HW/TL) of wild-type (WT) and TG mice at 5 months of age. HW: heart weight (mg); BW: body weight (g); TL: tibia length (mm); LW: liver weight (g) (n=3-6). The heart and liver were removed, weighed, and normalized to body weight or tibia length. *B*, Echocardiographic analysis of WT and TG hearts at 5 months of age (n=5-11). LVEDD: left ventricular end-diastolic dimension; LVESD: left ventricular end-systolic dimension; IVSTD: interventricular septal thickness in diastole; PWTD: posterior wall thickness in diastole; *P<0.05 (two-tailed Student’s *t*-test).

We then used rigorous and unbiased methods to check the relative heart weight (Figure 3A). By 5 months of age, the heart weight to body weight ratio (HW/BW) and heart weight to tibia length ratio (HW/TL) of MyD88 TG mice remained unchanged in lines B and D compared with those of age-matched WT mice. We used the liver weight to body weight ratio (LW/BW) and liver weight to tibia length ratio (LW/TL) as internal controls.

To clearly characterize physiological cardiac parameters of MyD88 TG mice, M-mode echocardiography was performed at 5 months of age (Figure 3B). Consistent with the heart weight data, no differences in left ventricular end-diastolic dimension (LVEDD), left ventricular end-stolic dimension (LVESD), interventricular septal thickness in diastole, and posterior wall thickness in diastole were observed in MyD88 TG mice, suggesting normal anatomy of MyD88 hearts.

### MyD88 transgenic mice developed reduced cardiac contractility at baseline

**Figure 4:**
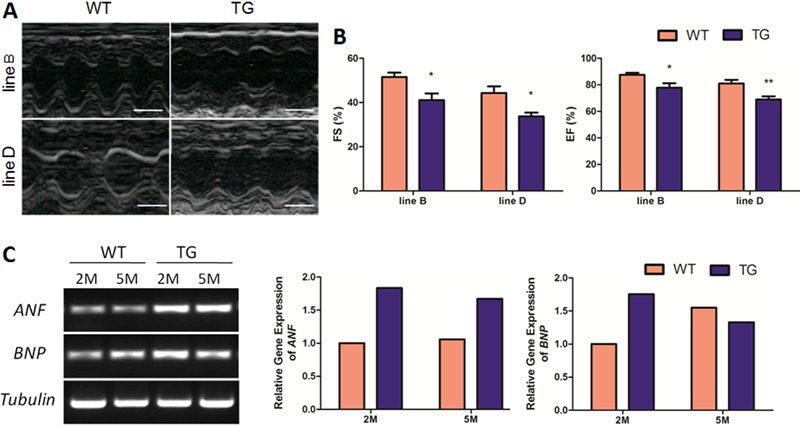
MyD88 transgenic (TG) mice showed reduced cardiac contractility and elevated heart failure marker expression. *A*, Representative M-mode echocardiographic views of wild-type (WT) and TG hearts at 5 months (M) of age. Scale bar, 20 μm. *B*, Statistical analysis of echocardiographic parameters of WT and TG mice (n=5-11). FS%: fractional shortening, FS% = (LVEDD−LVESD)/LVEDD×100; EF%: ejection fraction, EF% = (LVEDD^3^−LVESD^3^)/LVEDD^3^×100. *P<0.05, **P<0.01 compared to WT (two-tailed Student’s *t*-test). *C*, Measurement of atrial natriuretic factor (*ANF*) and brain natriuretic peptide (*BNP*) mRNA levels in WT and TG hearts at 2 and 5 months of age.

To directly determine cardiac contractility of MyD88 TG mice, echocardiography was performed on mice at the age of 5 months (Figure 4A). Fractional shortening (FS) is a popular echocardiographic index for left ventricular systolic contractility. As shown in Figure 4B, FS was reduced in MyD88 TG mice compared with WT mice (78.4%, P<0.05 for line B; 76.2%, P<0.05 for line D). Similarly, the ejection fraction, another index for left ventricular contractility, was also downregulated accordingly (88.0%, P<0.05 for line B; 85.2%, P<0.01 for line D).

Heart failure is closely associated with re-expression of fetal genes or upregulation of cardiac proteins, such as ANF and BNP (26). To more rigorously characterize the phenotype of MyD88 transgenic mice, mRNA expression of heart failure markers was performed. Consistent with the cardiac dysfunction phenotype, mRNA levels of *ANF* and *BNP* were elevated in MyD88 TG myocardium by 2 and 5 months of age compared with WT mice, which indicated that the molecular program for heart failure had been initiated (Figure 4C).

## Discussion

In this study, we analyzed a mouse strain with cardiac overexpression of MyD88 protein. We found that balanced MyD88 signaling maintained normal heart function. MyD88 TG mice developed mild cardiac dysfunction, without obvious abnormalities in cardiac morphology. This finding suggests a potential protective effect on the heart from excessive MyD88 expression.

Our study showed that excessive MyD88 did not trigger serious cardiac remodeling at the whole-organism level. In line with our observations, a previous study showed that transiently transfected MyD88 accumulates in granules and in larger, condensed structures throughout the nucleus and cytoplasm of HeLa cells (27). Consistent with these findings, Iliev et al. (28) reported that the ectopically expressed transgenic salmon MyD88 homolog (SsMyD88) accumulates in aggresomes, indicating that MyD88 might represent a defense mechanism against the potentially harmful effects of excessive MyD88 signaling. Therefore, unsurprisingly, excessive MyD88 did not trigger spontaneous extensive cardiac remodeling in our model.

A potential concern related to our study is that overexpression of a protein in the heart might induce a non-specific biological effect, resulting in cardiac dysfunction (29). However, more than one line of MyD88 transgenic mice demonstrated a similar cardiac dysfunction phenotype. This finding indicated that the observed phenotype was not related to overexpression-induced toxicity.

In summary, we generated a heart-specific MyD88 overexpression model to evaluate the potential harmful effect of excessive cardiac MyD88 signaling on pathogenesis of baseline cardiac remodeling. Our study shows that overexpression of MyD88 induced mild cardiac dysfunction without obvious abnormalities in heart morphology, suggesting a comprehensive role for MyD88 in cardiac remodeling responses.
